# Experimentally confirmed toltrazuril resistance in a field isolate of *Cystoisospora suis*

**DOI:** 10.1186/s13071-017-2257-7

**Published:** 2017-06-29

**Authors:** Aruna Shrestha, Barbara Freudenschuss, Rutger Jansen, Barbara Hinney, Bärbel Ruttkowski, Anja Joachim

**Affiliations:** 1 Institute of Parasitology, Department of Pathobiology, University of Veterinary Medicine Vienna, Vienna, Austria; 2 Boehringer Ingelheim, Alkmaar, The Netherlands

**Keywords:** Efficacy, Toltrazuril, Field isolate, Oocyst excretion, Pig

## Abstract

**Background:**

Constant treatment regimens with toltrazuril against *Cystoisospora suis* infection in piglets are being applied in the intensive production systems for the last two decades, but the possibility of resistance development has not been addressed so far despite limited availability of treatment alternatives. Recently, a pig producer in The Netherlands who routinely used toltrazuril complained about diarrhea in suckling piglets in the absence of bacterial and viral pathogens, and oocysts of *C. suis* could be isolated from feces of affected litters.

**Methods:**

Piglets from two litters were infected with a field isolate of *C. suis*, Holland-I, and treated with 0 (Holl-Ctrl), 20 (Holl-20) or 30 (Holl-30) mg/kg of body weight (BW) of toltrazuril (Baycox®). The efficacy of toltrazuril was measured by assessment of oocyst excretion, fecal consistency and BW gain. A separate litter was infected with a toltrazuril-susceptible strain of *C. suis*, Wien-I, and treated with 0 (Wien-Ctrl) or 20 (Wien-20) mg/kg BW of toltrazuril for comparison.

**Results:**

Treatment with the recommended (20 mg/kg) dose of toltrazuril completely suppressed oocyst shedding and diarrhea in group Wien-20. The prevalence of oocyst excretion was 100% in the groups infected with Holland-I and 80% in the group Wien-Ctrl. Most days with diarrhea were observed in group Holl-20 with an average of 6.40%, followed by 5.71% in Wien-Ctrl, while in Holl-Ctrl and Holl-30 diarrhea was only seen in 1.79% of the samples (*n* = 14/piglet). Oocyst excretion, fecal consistency and BW gain did not differ significantly among groups infected with Holland-I, indicating loss of efficacy to toltrazuril.

**Conclusion:**

Experimental infections and treatment confirmed toltrazuril resistance against the field isolate even at increased dosage. Such isolates are a potential threat to pig production as no other effective and economically sustainable alternative treatment is currently available. In the absence of a standardized protocol for resistance testing in *C. suis*, regular parasitological examination and, if possible, experimental confirmation should be considered to evaluate the extent and consequences of toltrazuril resistance.

**Electronic supplementary material:**

The online version of this article (doi:10.1186/s13071-017-2257-7) contains supplementary material, which is available to authorized users.

## Background

Coccidiosis is a major parasitic disease affecting a wide range of livestock and wild animals globally. In pigs, *Cystoisospora suis* (syn. *Isospora suis*) is the most pathogenic species of swine coccidian and most severely affects suckling piglets [1–4]. Clinical signs include pasty to watery non-hemorrhagic diarrhea, weight loss and ill thrift [5–10]. At present, cystoisosporosis is considered as one of the leading causes of diarrhea in neonatal piglets with high prevalences worldwide [3, 7, 11–14]. Oocysts are highly resistant to desiccation and antimicrobial compounds [14, 15] making elimination virtually impossible once they have been introduced into the farm. The disease shows a very high morbidity with low mortality, and not all the piglets in a litter are equally affected, resulting in reduced, uneven weaning weights and thus, ultimately in often significant economic losses [5, 11, 16, 17].

In the European Union (EU), control of cystoisosporosis is commonly achieved with a single oral administration of toltrazuril in the prepatent period (day 3–5 of life). Baycox® is the trade name of a broad spectrum anticoccidial drug containing the triazine trione toltrazuril [18]. Use of Baycox® in piglet coccidiosis was first authorized in Australia in 1998 to be administered once in the first week of life at a dose of 20 mg/kg body weight (BW) [19]. A single oral treatment with toltrazuril administered during the prepatent period provided effective and sustained suppression of oocyst shedding and diarrhea in piglets experimentally infected with *C. suis* [17, 20–22] and under field conditions [23–28]. However, emerging resistance in poultry coccidia against anticoccidials including toltrazuril is of growing concern [29–31]. Constant treatment regimens with toltrazuril have been applied for controlling porcine cystoisosporosis in the EU for almost two decades now, but the possibility of resistance development in *C. suis* isolates has not been addressed so far, despite limited availability of treatment alternatives. In 2014, a pig farmer in The Netherlands, with a farrow-to-finish herd of 330 sows, complained about pasty feces in 60% of the piglets from 10 days of age until weaning despite treatment with the recommended dose of toltrazuril. The involvement of bacterial and viral pathogens was excluded as the possible pathogens associated with piglets’ diarrhea could neither be re-isolated nor be detected by polymerase chain reaction (PCR). Presence of villous atrophy and fusion in the histological sections of jejunum indicated cystoisosporosis [32, 33], which was later confirmed by the detection and isolation of *C. suis* oocysts in the fecal samples. Evaluation of the administered amount of toltrazuril on the farm level revealed no under-dosing. Moreover, application of twice the recommended dose (40 mg/kg) of toltrazuril also did not have any effect on the clinical picture and thus loss of efficacy was suspected. In the present study, the efficacy of toltrazuril against *C. suis* infections in suckling piglets was evaluated in experimental infections with the mentioned field isolate, Holland-I, and a toltrazuril-sensitive strain, Wien-I. In the past, experimental studies have indicated development of resistance under field conditions in *Eimeria* of poultry [34–36]. To our knowledge, this is the first report of experimentally confirmed toltrazuril resistance in a field isolate of *C. suis*.

## Methods

### Study animals

A total of 34 conventionally raised healthy piglets from three crossbred sows (Landrace × Large White) were allotted to five treatment groups (Table 1). Sows were housed on straw in individual farrowing crates in the animal husbandry facility of the Institute of Parasitology, University of Veterinary Medicine Vienna, Austria. All rooms were equipped with daylight and ventilation, and room temperature of 18–20 °C was maintained throughout the trial. Fresh drinking water was provided ad libitum to the sows and piglets. The sows were fed once daily with a commercial feed without coccidiostat according to the manufacturer’s recommendation and the piglets received milk from the sow followed by starter feed from the second week of life. The day of birth of piglets was considered as study day 1 (SD 1). All piglets were ear-marked and received 100 mg iron dextran on SD 2 to prevent iron deficiency. The sows arrived two weeks prior to the expected parturition date for adaptation to the new environment.

**Table 1:** Groups, infections and treatment in the trial. Infection: study day 4; treatment: toltrazuril (Baycox® 5% suspension for piglets) once on study day 6

Group	*C. suis* strain	Litter no.	Treatment/Dose	No. of piglets
Wien-Ctrl	Wien-I	1	Tap water; 1 ml	5
Wien-20	Wien-I	1	Toltrazuril; 20 mg/kg body weight	5
Holl-Ctrl	Holland-I	2	Tap water; 1 ml	4
		3		4
Holl-20	Holland-I	2	Toltrazuril; 20 mg/kg body weight	4
		3		4
Holl-30	Holland-I	2	Toltrazuril; 30 mg/kg body weight	4
		3		4

### Study design

The clinical trial followed a blinded and incompletely randomized block design consisting of two blocks (one for each *C. suis* strain), each containing the control and the treatment group(s). The experimental unit was the individual animal. Randomization was carried out in each block assigning piglets to the respective treatment group (*n* = 5 to 8 piglets/group), ranking animals based on decreasing birth weight. The animals were distributed among the litters as described in Table 1.

### Parasite material and experimental infection

Oocysts of the Holland-I field isolate of *C. suis* were obtained from fecal samples originating from the mentioned commercial farm in The Netherlands with suspected reduced sensitivity to toltrazuril. Before performing resistance studies the field isolate was passaged once through piglets for collection of fresh, viable oocysts. A toltrazuril-sensitive strain of *C. suis*, Wien-I [20], was used for comparison between the strains which was maintained and passaged regularly in suckling piglets for the production of infectious oocysts at the Institute of Parasitology, University of Veterinary Medicine Vienna, Austria. The strain is passaged every 3–6 months and infectivity in vivo and susceptibility to toltrazuril are assessed regularly. Each piglet was inoculated orally with 1000 *C. suis* oocysts, suspended in 1 ml of tap water, of the respective *C. suis* strain on SD 4 using a flexible plastic Pasteur pipette. The groups were denominated on the basis of treatment (Table 1) on SD 6 and received either sham-treatment or a commercial formulation of toltrazuril (Baycox® 5% oral suspension; Batch no: KP0BFX9; Expiry date: 06/2021, Leverkusen, Germany). Animals infected with Wien-I received the recommended dose of 20 mg/kg of body weight (BW) of toltrazuril (Wien-20). Piglets infected with Holland-I were treated with 20 mg/kg BW (Holl-20) or an elevated dose of 30 mg/kg BW (Holl-30) of toltrazuril. Piglets in the sham-treated control groups (Wien-Ctrl and Holl-Ctrl) received 1 ml of tap water orally. The efficacy of toltrazuril was evaluated by assessment of body weight development, fecal consistency and oocyst excretion.

### Evaluation of fecal samples

Individual fecal samples were collected daily from SD 8 to 21 for the evaluation of fecal consistency and oocyst excretion. Fecal consistency was scored immediately after sampling according to the following key: fecal score (FS) 1 = normal; FS 2 = pasty; FS 3 = semi-liquid; and FS 4 = liquid, with FS 3 and FS 4 considered as diarrhea [7]. Fecal samples were first screened for oocysts by autofluorescence (AF) detection under UV light [37] with a sensitivity of ca. 10 OpG; in positive samples oocyst excretion was determined quantitatively using a modified McMaster technique [3].

### Body weight and general health observation

The piglets were weighed on SD 1, 8, 15 and 22. Additionally, the body weight of each piglet was recorded on the day of treatment for calculation of the treatment dose. All piglets were observed daily during the course of the studies to ensure good general health and any condition that required veterinary care was recorded and addressed.

### Differential diagnosis

Pooled fecal samples of each litter were screened on SD 8 for the presence of any other pathogens causing diarrhea in neonatal piglets including rotavirus, coronavirus, *E. coli* and *C. perfringens*.

### Statistical analysis

Statistical calculations were performed with RStudio version 0.99.896 (RStudio Team, 2016), descriptive statistics with Microsoft Excel 2010 and GraphPad Prism version 5.04 for Windows (GraphPad Software, San Diego, California USA). Differences in clinical and parasitological parameters between groups were analyzed applying an ANOVA in case of normal distribution and variance homogeneity of the data, or a Kruskal-Wallis rank sum test if this was not the case. In the event of significance for the omnibus tests parametric or non-parametric *post-hoc* tests for multiple comparisons were performed (according to Tukey and Conover, respectively), using *P*-value adjustment after Bonferroni. Statistical calculations were restricted to groups Holl-Ctrl, Holl-20 and Holl-30 (*n* = 8 animals/group) due to the small size of groups Wien-Ctrl and Wien-20 (*n* = 5 animals/group). Spearman’s rank correlation coefficient was calculated to describe the relationship between selected parameters. *P*-values ≤ 0.05 were considered significant.

## Results

### Oocyst excretion

**Fig. 1:**
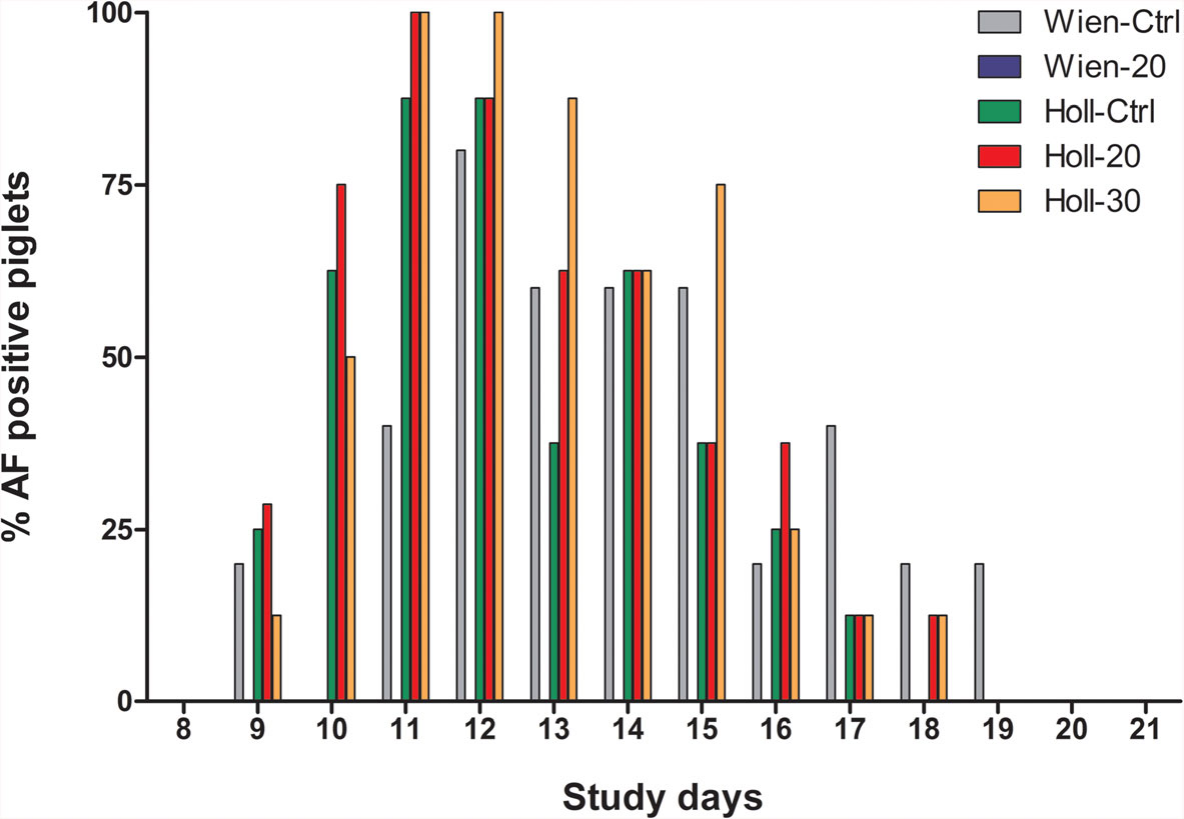
Percentage of *C. suis* positive piglets on the respective study days as detected by fluorescence microscopy

**Fig. 2:**
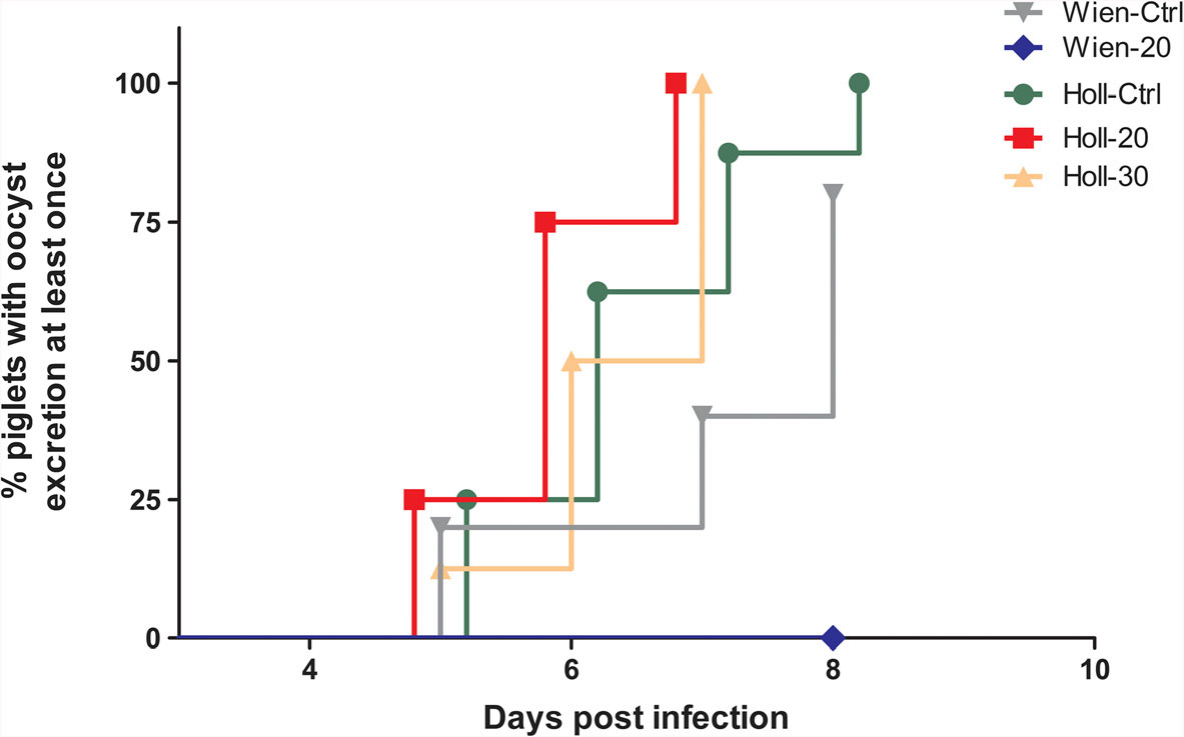
Percentage of piglets with oocyst excretion at least once post-infection as detected by fluorescence microscopy

Excretion of *C. suis* oocysts was completely suppressed by the treatment in group Wien-20 while all other groups excreted oocysts detectable in AF (Fig. 1) and McMaster (Table 2) techniques. Oocyst shedding was first observed in these groups on SD 9, and by SD 12 all animals except one had been positive at least once (Fig. 2). In groups Holl-Ctrl, Holl-20 and Holl-30 every piglet excreted oocysts at least once, whereas in group Wien-Ctrl all piglets except one shed oocysts (Table 2). The prevalence in piglets infected with Holland-I peaked on SD 11 with 87.5% positive piglets in group Holl-Ctrl and 100% positive piglets in groups Holl-20 and Holl-30. In group Wien-Ctrl the prevalence reached its maximum (80%) on SD 12 (Fig. 1). Prepatency tended to be shorter in piglets infected with Holland-I (Table 2). The number of excretion days (AF) was similar in all groups with excretion, ranging from 30.0 to 38.4% of all sampling days in the different groups (Table 2).

**Table 2:** Overview of the parasitological parameters (AF and McMaster countable excretion) for all experimental groups

Group	% piglets with oocyst excretion	Mean ± SD excretion days/piglet	% excretion days ± SD (14 sampling days/piglet)	% days with McMaster countable excretion ± SD	Mean ± SD prepatency (days)	Mean AUC Study days 9–19	Mean ± SD OpG Study days 9–19	Median OpG Study days 9–19	Max OpG (Study day)
Wien-Ctrl	80	4.2 ± 3.0	30.0 ± 21.1	27.1 ± 23.4	7.0 ± 1.4	8,892.8	12,902.2 ± 12,950.8	2,997.0	97,236 (11)
Wien-20	0	0	0	–	–	–	–	–	–
Holl-Ctrl	100	4.4 ± 2.1	31.3 ± 15.2	25.9 ± 14.8	6.3 ± 1.0	16,444.8	23,169.9 ± 29,227.4	666.0	365,301 (14)
Holl-20	100	5.0 ± 1.8	36.3 ± 12.4	23.4 ± 15.0	6.0 ± 0.8	19,450.9	21,723.1 ± 21,261.8	1,332.0	166,500 (9)
Holl-30	100	5.4 ± 1.9	38.4 ± 13.2	32.1 ± 15.3	6.4 ± 0.7	22,193.6	18,976.4 ± 15,221.2	1,998.0	196,803 (9)

For parameters related to quantitative excretion, only the period with observed McMaster counts was evaluated

*Abbreviations*: *AUC* area under the curve for OpG (oocysts per gram of feces), *SD* standard deviation

**Fig. 3:**
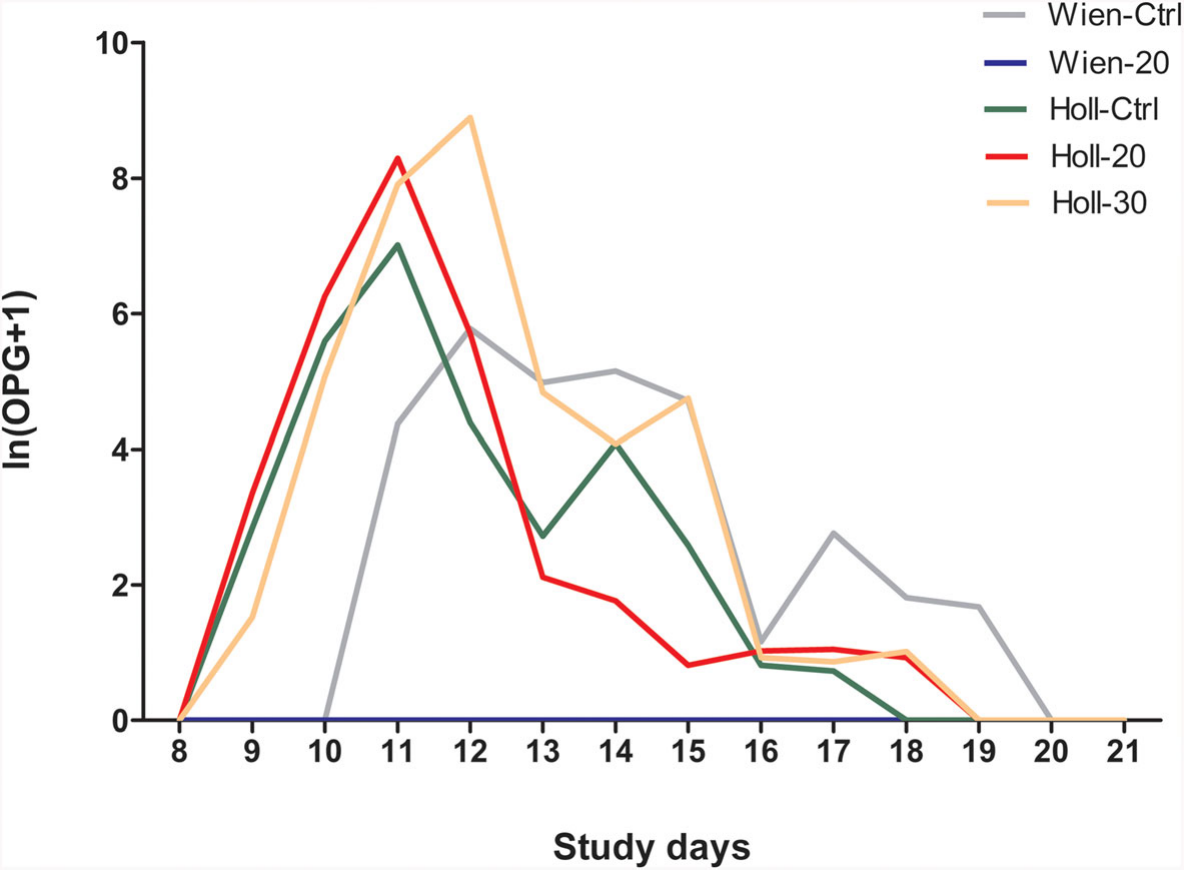
Daily oocyst excretion (ln (OpG + 1)) in piglets from study days 8–21 as determined by McMaster technique

The highest individual oocyst shedding (max OpG) was seen in group Holl-Ctrl on SD 14. Generally, max OpGs were higher in groups infected with the Holland-I isolate (Table 2). Oocyst excretion reached its peak on SD 11–12 (Fig. 3), and was most pronounced in group Holl-30 with a mean OpG value of 196,803. The amount of shed oocysts exhibited high variabilities between individuals, ranging from 333 to 365,301 within the same group, without significant differences in daily mean OpG values, the area under the curve (AUC) for OpG SD 9 to 19 as well as the mean OpG SD 9 to 19 between groups infected with Holland-I.

### Fecal consistency and diarrhea

**Fig. 4:**
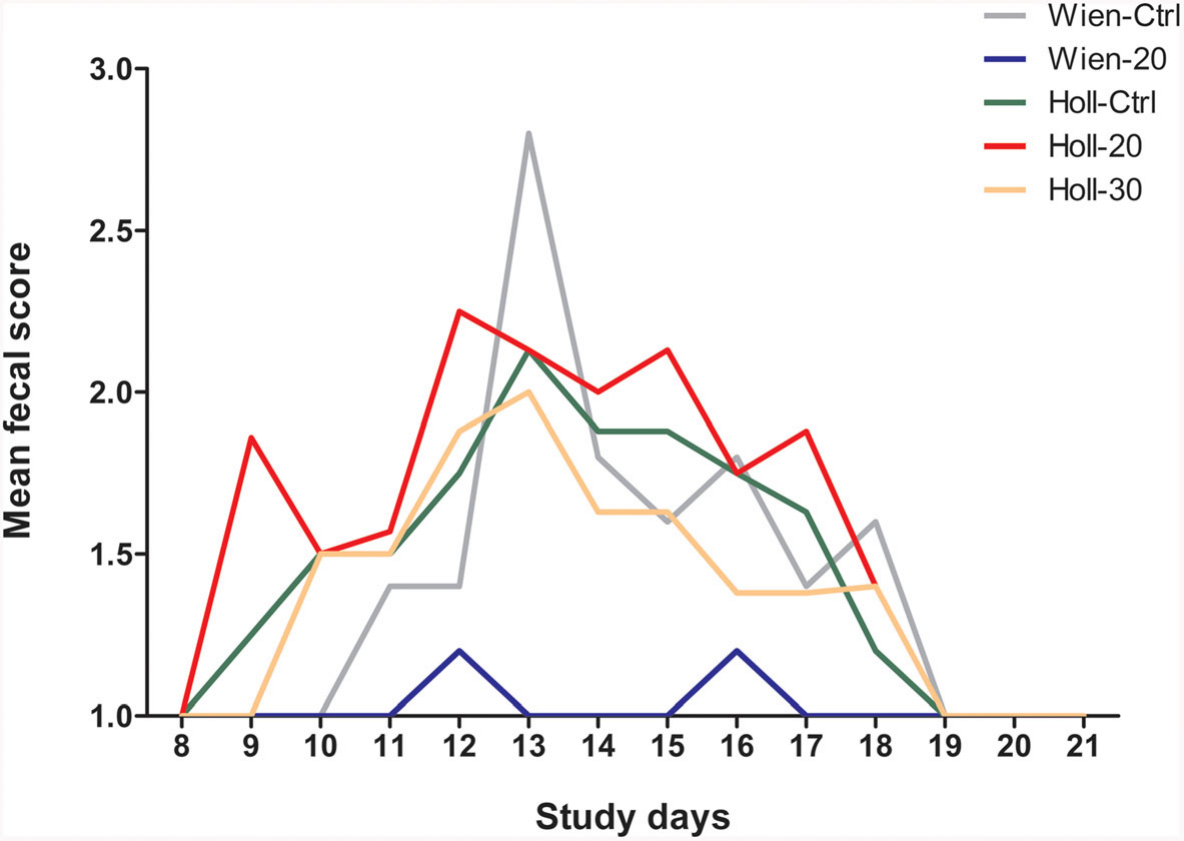
Mean fecal scores of piglets throughout the sampling period (study days 8–21)

None of the study animals had diarrhea on the day of infection. Moreover, the mean fecal score of Wien-20 did not exceed 1.20 throughout the study. The mean FS reached its peak on SD 13 in groups Wien-Ctrl, Holl-Ctrl and Holl-30 with 2.80, 2.13 and 2.00, respectively, and with 2.25 on SD 12 in group Holl-20 (Fig. 4). Neither the overall mean FS from SD 9 to 18 (Table 3) nor the mean FS on single study days differed significantly between groups infected with Holland-I.

**Table 3:** Clinical parameters in the forms of fecal consistency and intensity of diarrhea in all experimental groups over the study period

Group	% piglets with diarrhea at least once	% diarrhea days ± SD (14 sampling days/piglet)	Mean ± SD fecal score (study days 9–18)
Wien-Ctrl	60.0	5.7 ± 6.0	1.6 ± 0.4
Wien-20	0	0	1.0 ± 0.1
Holl-Ctrl	25.0	1.8 ± 3.3	1.7 ± 0.4
Holl-20	62.5	6.4 ± 6.0	1.9 ± 0.3
Holl-30	12.5	1.8 ± 5.1	1.5 ± 0.3

For quantitative evaluation of the fecal score only the period of mean values >1.00 was evaluated for study days 9–18

Diarrhea was not observed at all in group Wien-20. The prevalence of diarrhea peaked on SD 13 in groups Wien-Ctrl and Holl-Ctrl with 60 and 25% of the piglets having FS > 2, respectively. In group Holl-20, the prevalence reached its maximum already on SD 9 (28.57%) and on SDs 12 and 13 in group Holl-30 with 12.5% each. Most days with diarrhea were observed in group Holl-20 with an average of 6.40%, followed by 5.71% in group Wien-Ctrl, while in groups Holl-Ctrl and Holl-30 diarrhea was only seen in 1.79% of the samples. Percentage of piglets with FS > 2 at least once ranged from 12.5% (Holl-30) to 62.5% (Holl-20) (Table 3). However, the number of diarrhea days and piglets that had diarrhea at least once did not differ significantly between groups infected with Holland-I. FS 4 (watery) was only observed in two piglets of group Wien-Ctrl on SD 13.

### Body weight

Since the birth weight of two individual piglets was below 0.9 kg, they were excluded from the body weight calculation. Mean body weights and mean body weight gains (baseline: SD1) did not differ significantly (*P* > 0.05) between groups Holl-Ctrl, Holl-20 and Holl-30 throughout the study (Additional file 1: Table S1) although a significant negative correlation between the mean fecal score and the individual weight gain from SD 1 to 22 was observed for all included animals (*ρ* = -0.486, *P* = 0.005).

### Differential diagnosis

Fecal samples pooled by litter on SD 8 tested negative for rotavirus and coronavirus while *E. coli* as well as *C. perfringens* could be isolated from all litters.

### General health

Piglets showed softened feces (FS 2) or diarrhea (FS 3 or 4) as described. No condition related to the experimental infection requiring veterinary treatment was observed.

## Discussion

Resistance to all classes of anticoccidials is well described in avian coccidia [30, 38, 39]. Despite the long-term use of toltrazuril in piglet production and the lack of satisfying treatment alternatives, the possibility of resistance development has not been addressed so far. In the present study we evaluated the efficacy of toltrazuril against a *C. suis* isolate from a Dutch farm complaining about symptoms typical of coccidiosis despite metaphylactic treatment with the recommended dose of toltrazuril. In an experimental setup, two litters were infected with the isolate in question and treated with 0, 20 or 30 mg/kg BW of toltrazuril. Additionally, a third litter was infected with a toltrazuril-sensitive strain (Wien-I) and treated with 20 mg/kg BW of toltrazuril or sham treated to confirm effectiveness of the Baycox® batch used. Due to animal welfare reasons only a minimum number of animals were infected with this strain; therefore, statistics could not be employed for these groups. However, the positive effect of toltrazuril on oocyst excretion, fecal score and body weight gain was sufficiently shown in several earlier field and experimental studies [7, 17, 20, 21, 24, 26, 40, 41]. It completely inhibits the development of all parasitic stages of *C. suis* and, given during the prepatent period of infection, prevents tissue damage and consequently the emergence of diarrhea [42].

Resistance is described by the World Health Organization [43] as the “ability of a parasite strain to survive and/or multiply despite the administration and absorption of a drug given in doses equal to or higher than those usually recommended but within tolerance of the subject”. This definition can also be employed for coccidia. The reliability of toltrazuril to significantly reduce the excretion of *C. suis* oocysts was already shown in a number of experimental studies [20, 21, 41, 42]. Mundt et al. [7] described a complete suppression of oocyst shedding in experimentally infected and toltrazuril-treated animals while every untreated animal shed parasites at least once. In another study conducted by Mundt et al. [17], the treatment with toltrazuril resulted in significantly fewer mean excretion days (0.6 *vs* 4.0), fewer piglets with oocyst excretion (30 *vs* 91%) and a significantly lower mean OpG (144 *vs* 17,797) 5 to 11 days post-infection (dpi). This is in accordance to a field study conducted by Kreiner et al. [24] where the number of *C. suis* positive samples was significantly lower in toltrazuril-treated compared to untreated piglets in different herds. The same effect was observed in this study for group Wien-20, where oocyst shedding was completely suppressed by the treatment. By contrast, treatment with toltrazuril did not impair oocyst development and excretion in the groups infected with the Dutch isolate, Holland-I. In avian coccidiosis complete drug resistance is defined by ineffectiveness despite higher doses [44]. Every single piglet infected with the Holland-I isolate shed oocysts regardless of the toltrazuril dose. Moreover, the number of AF countable excretion days, the AUC for OpG, the maximum individual OpG as well as the mean OpG from SD 9 to 19 was comparable between the control and treatment groups of this isolate, indicating complete drug resistance. The above mentioned parameters tended to be higher in groups Holl-Ctrl, Holl-20 and Holl-30 compared to group Wien-Ctrl, and the prepatent period for excretion detected by AF technique was shorter in Holland-I compared to Wien-I. Variations in prepatent periods can be attributed not only to factors such as infection dose or age and health of piglets (which were comparable between all groups) but also to the virulence of *C. suis* isolates [7, 45, 46]. However, as oocyst excretion underlies large individual variations [7, 47–49] reliable conclusions about possible differences between strains regarding this particular trait cannot be drawn at this point in time.

While mean fecal consistencies remained below 1.2 in group Wien-20, all other groups developed increased mean fecal scores after infection, indicating enteritis as a consequence of parasite replication [50]. Diarrhea was observed in all groups except group Wien-20, but prevalences and numbers of diarrhea days were generally low. It has been previously shown that toltrazuril successfully reduces fecal scores and suppresses diarrhea in piglets infected with *C. suis* [17, 41, 42, 51]. In a field study, Scala et al. [26] found the overall diarrhea prevalence to be significantly lower in toltrazuril-treated animals compared to untreated animals. Similarly, Kreiner et al. [24] found treated animals to have a significantly lower mean FS and significantly fewer diarrheic fecal samples in the field. In two trials conducted by Joachim & Mundt [20] not a single piglet with diarrhea was observed after treatment with toltrazuril while almost all untreated animals had a FS of 3 or 4 (85.7 and 100%, respectively) at least once. This was also described by Mundt et al. [7] who observed an average FS of 2 or more in experimentally infected piglets throughout the study, with daily diarrhea prevalences between 25 and 75%. On the other hand, the mean FS of animals treated with toltrazuril remained between 1 and 2 during the entire sampling period. In this study, treatment had obviously no effect on fecal consistency of the piglets infected with the Dutch isolate. The group Holl-20 showed the highest mean fecal score, the highest prevalence of diarrhea and most diarrhea days among the three groups. These parameters were also similar in groups Holl-Ctrl and Holl-30, indicating complete lack of clinical efficacy of toltrazuril. Despite the limited data set available so far for Holland-I, this strain might be of low virulence since overall prevalences and days of diarrhea were low and a FS of 4 was not observed even once. However, inter-and intra-litter deviations cannot be excluded in this setting. Just as with oocyst excretion, the development and severity of diarrhea varies between litters and individuals [48, 52, 53] and is also influenced by other factors [7].

Chapman [54] considered an *Eimeria* strain to be resistant if the weight gain of treated infected chicken did not differ significantly from that of untreated infected controls. In fact, treatment with the recommended or the elevated dose did not have a significant effect on body weight gains in the Dutch isolate, indicating resistance to toltrazuril. This finding is not surprising as the mean fecal score, which was negatively correlated with the individual weight gain, did not differ significantly between the control and treatment groups of isolate Holland-I. It has previously been shown that diarrhea is negatively correlated with the body weight gain in *C. suis* infections and that the application of toltrazuril significantly increases the weight gain of piglets compared to untreated infected controls [7, 17, 26, 40], although for the groups infected with Wien-I this could not be shown due to the small group size.

On grounds of the clinical picture observed during the study, it can be assumed that the bacterial agents identified by microbiological examination are facultative pathogenic subtypes regularly found in porcine intestinal flora [55, 56]. This is underpinned by the fact that no sign of disease occurred in group Wien-20, despite the presence of the same bacteria as in all other groups. Jonach et al. [57] did not find statistical differences in the intestinal abundance of *E. coli* or *C. perfringens* when comparing piglets with and without diarrhea. This was confirmed by Ruiz et al. [58] who found similar prevalences in piglets with diarrhea and control piglets while frequencies of *C. suis* were significantly higher in diarrheic piglets.

To our knowledge, this study is the first to experimentally confirm toltrazuril resistance in a *C. suis* isolate. There are currently no satisfying treatment alternatives available [20, 23], underlining the need of new intervention strategies against porcine neonatal coccidiosis. Sulfonamides have been suggested against porcine coccidiosis but a short-term oral administration was shown to have an unsatisfying effect [17, 26]. Only an injectable sulfonamide, repeatedly administered for 6 to 7 days, had an effect on parasite development and clinical outcome comparable to that of toltrazuril under experimental conditions [20]. Such a treatment may be considered as an alternative in cases of toltrazuril resistance, but it is labor-intensive and unsuited for routine treatment. Therefore, alternative control strategies to chemometaphylaxis are desirable [59].

Luckily, it seems that resistance in *C. suis* develops far slower than in avian coccidia. Vertommen et al. [60] described the development of resistance to Baycox® against *Eimeria* on a broiler farm within four fattening periods. Nevertheless, the rise of further resistant *C. suis* isolates may just be a matter of time as the extensive use of a drug over a longer period of time will inevitably lead to decreased efficacy [29, 44]. Currently, the sensitivity of *C. suis* isolates to toltrazuril can only be evaluated *in vivo*. A less laborious and faster *in vitro* assay, possibly employing the already established cell culture system [61], would be advantageous.

## Conclusions

Toltrazuril resistant *C. suis* isolates are a potential threat to pig farming as no other effective and economically sustainable alternative treatment is available. All piglets infected with the field isolate of *C. suis* Holland-I showed appreciable levels of diarrhea and oocyst excretion unresponsive to treatment. Therefore, veterinarians and farmers should be aware of the possibility of resistance development with long-term application of toltrazuril in intensive piglet production systems. In the absence of vaccines and effective anticoccidial agents other than toltrazuril, routine fecal screening and periodical assessment of efficacy of toltrazuril must be considered essential for the sustainable control of cystoisosporosis. In cases of reduced efficacy optimized hygiene measures employing regular chemical disinfection with a cresol-based product must be enforced.

## Additional file

Additional file 1: Table S1. Mean body weights and body weight gains in grams with standard deviations in brackets. SD: Study day. (DOCX 15 kb)

## Abbreviations

AF: Autofluorescence
AUC: Area under the curve
BW: Body weight
dpi: Days post-infection
EU: European Union
FS: Fecal score
OpG: Oocysts per gram feces
SD: Study days
