# Supplementary material for: Experimentally confirmed toltrazuril resistance in a field isolate of Cystoisospora suis
Source: Parasit Vectors. 2017 Jun 29;10:317. doi: 10.1186/s13071-017-2257-7 (PMC5492287; doi:10.1186/s13071-017-2257-7)
Supplement: Additional file 1: Table S1. — Mean body weights and body weight gains in grams with standard deviations in brackets. SD: Study day. (DOCX 15 kb) [file 13071_2017_2257_MOESM1_ESM.docx]

**Additional file 1: Table S1.** Mean body weights and body weight gains in grams with standard deviations in brackets. SD: Study day

| **Group** | **SD 1** | **SD 6** | **SD 8** | **SD 15** | **SD 22** | **Mean body weight gain SD 1 - 22** | **% body weight gain**  **SD 1 - 22** |
| --- | --- | --- | --- | --- | --- | --- | --- |
| **Wien-Ctr** | 1,310.0 [294.5] | 2,120.0 [317.4] | 2,640.0 [334.3] | 4,200.0 [443.0] | 6,250.0 [703.6] | 4,940.0  [490.4] | 477.1 |
| **Wien-20** | 1,425.0 [225.5] | 2,225.0 [440.6] | 2,425.0 [970.0] | 3,975.0 [673.9] | 6,187.5 [852.8] | 4,762.5  [683.6] | 434.2 |
| **Holl-Ctrl** | 1,412.5 [232.6] | 2,112.5 [393.5] | 2,743.8 [608.5] | 3,837.5 [800.3] | 5,650.0 [901.6] | 4,237.5  [715.5] | 400.0 |
| **Holl-20** | 1,450.0 [223.6] | 2,228.6 [232.5] | 2,757.1 [416.8] | 3,764.3 [732.4] | 5,571.4 [899.0] | 4,121.4  [701.7] | 384.2 |
| **Holl-30** | 1,456.3 [259.7] | 2,293.8 [217.8] | 3,112.5 [486.1] | 4,100.0 [504.3] | 5,937.5 [599.3] | 4,481.3  [414.9] | 407.7 |
